# What should the C (‘congestive heart failure’) represent in the CHA_2_DS_2_‐VASc score?

**DOI:** 10.1002/ejhf.1946

**Published:** 2020-07-22

**Authors:** Bart A. Mulder, Dirk J. van Veldhuisen, Michiel Rienstra

**Affiliations:** ^1^ University of Groningen, University Medical Center Groningen Groningen The Netherlands

## Introduction

Patients with atrial fibrillation (AF) require anticoagulation therapy when at least two clinical risk factors for stroke or thromboembolism are present, as defined in the CHA_2_DS_2_‐VASc score.^1^ In this score the C stands for ‘congestive heart failure’ and nowadays the criteria to qualify for a C in clinical practice are more or less synonymous to the presence of signs/symptoms of heart failure.^1^ The criteria for the CHA_2_DS_2_‐VASc score were, however, defined and developed at a time when heart failure was more or less restricted to patients who had left ventricular systolic dysfunction [or reduced left ventricular ejection fraction (LVEF)]. Whether these criteria also apply to patients who have heart failure with preserved ejection fraction (HFpEF), i.e. whether the C in the CHA_2_DS_2_‐VASc score also ‘counts’ in this population, is however unknown.^2^ This is of interest as an increasing proportion of patients with heart failure have HFpEF, AF is more common in HFpEF and these patients have a similar increased risk for stroke or cardiovascular events.^3^, ^4^, ^5^ Diagnosing HFpEF has been increasingly important but remains challenging as compared to diagnosing a heart failure with a reduced ejection fraction (HFrEF). This viewpoint will focus on the history of the C in the CHA_2_DS_2_‐VASc score and why it may or may not be considered to extrapolate the CHA_2_DS_2_‐VASc criteria to patients with HFpEF and AF as well.

## History of the C in the CHA_2_DS_2_‐VASc score

In patients with AF, who have a CHA_2_DS_2_‐VASc risk score of ≥2 (points) in men, and ≥3 (points) in women, anticoagulation therapy is generally recommended (class IA recommendation in the European Society of Cardiology AF management guidelines).^1^ The clinical characteristics from which the CHA_2_DS_2_‐VASc score is derived are, however, all based on registry data.^6^ This is important to realize, since the definition for heart failure has evolved over recent years.^3^ First, the term generally used nowadays is no longer ‘congestive’ heart failure, but rather ‘chronic’ heart failure, which is related to the fact that not all patients have obvious signs of congestion and also the distinction is primarily made between acute and chronic heart failure. Second, and more importantly, since 2016 heart failure is categorized into three groups based on LVEF: reduced (<40%), mid‐range (40–49%) and preserved (>50%). In the first description of the CHADS_2_ score, the precursor of the CHA_2_DS_2_‐VASc score, the C was classified as recent (i.e. in the last 100 days in one of the studies^7^) congestive heart failure exacerbation (without a LVEF criterium).^8^ The CHA_2_DS_2_‐VASc score is based on the CHADS_2_ score and uses the same definitions. In the first paper by Lip *et al*.^6^ proposing the CHA_2_DS_2_‐VASc score, the Euro Heart Survey was used as a validation cohort, where congestive heart failure was classified as ‘heart failure’ or ‘left ventricular ejection below 35%’. The group ‘heart failure’ in that study is possibly reflecting patients with symptoms of heart failure, with and without reduced ejection fraction, so it may be suggested that also HFpEF patients were included, although these data are not reported.

## Stroke prediction risk scores in atrial fibrillation

The CHADS_2_ and CHA_2_DS_2_‐VASc scores were not the first (and not the last) attempts for a reliable stroke prediction risk score in AF.^9^ The CHADS_2_ score was the result of previous risk scoring models, namely the Atrial Fibrillation Investigators (AFI) scheme and the Stroke Prevention and Atrial Fibrillation (SPAF) scheme.^7^ In the AFI risk scheme, data were collected from five other trials: (i) the Atrial Fibrillation, Aspirin, Anticoagulation Study from Copenhagen, Denmark (AFASAK), (ii) the Stroke Prevention in Atrial Fibrillation (SPAF) study, (iii) the Boston Area Anticoagulation Trial in Atrial Fibrillation (BAATAF), (iv) the Canadian Atrial Fibrillation Anticoagulation (CAFA) study, and (v) the Veterans Affairs Stroke Prevention in Nonrheumatic Atrial Fibrillation (SPINAF) study. Congestive heart failure was considered as a risk factor, but was not qualified similarly amongst the studies (see *Table* *1* for an overview of the studies). For example, in the AFASAK trial only patients with symptomatic moderate and severe heart failure were considered to have congestive heart failure.^10^ Notably, no data on LVEF were provided in any of these trials and it is uncertain what type of heart failure these patients really had (reduced, mid ranged or preserved LVEF). It appears, however, that from a historical perspective, many of these patients must have been HFrEF patients.^3^ In conclusion, in the original cohorts, predominantly HFrEF patients were included as HFpEF was not acknowledged at that time. Therefore, the C of congestive heart failure, appears to be primarily driven by HFrEF.

**Table 1:** Overview of the definition of heart failure as the basis for the C in CHA_2_DS_2_‐VASc

Study	Definition of ‘heart failure’ in this study	LVEF of heart failure patients	Incorporated in AFI, CHADS_2,_ CHA_2_DS_2−_VASc
AFASAK	Symptoms of heart failure (non specified)	Not reported	Yes
SPAF	History of congestive heart failure	Not reported	Yes
BAATAF	History of congestive heart failure	Not reported	Yes
CAFA	Heart failure (non specified)	Not reported	Yes
SPINAF	History of congestive heart failure	Not reported	Yes

AFASAK, Atrial Fibrillation, Aspirin, Anticoagulation Study from Copenhagen, Denmark; AFI, Atrial Fibrillation Investigators; BAATAF, Boston Area Anticoagulation Trial in Atrial Fibrillation; CAFA, Canadian Atrial Fibrillation Anticoagulation; LVEF, left ventricular ejection fraction; SPAF, Stroke Prevention in Atrial Fibrillation; SPINAF, Veterans Affairs Stroke Prevention in Nonrheumatic Atrial Fibrillation.

## Pathophysiology of atrial fibrillation‐related stroke in heart failure with preserved and reduced ejection fraction

**Figure 1:**
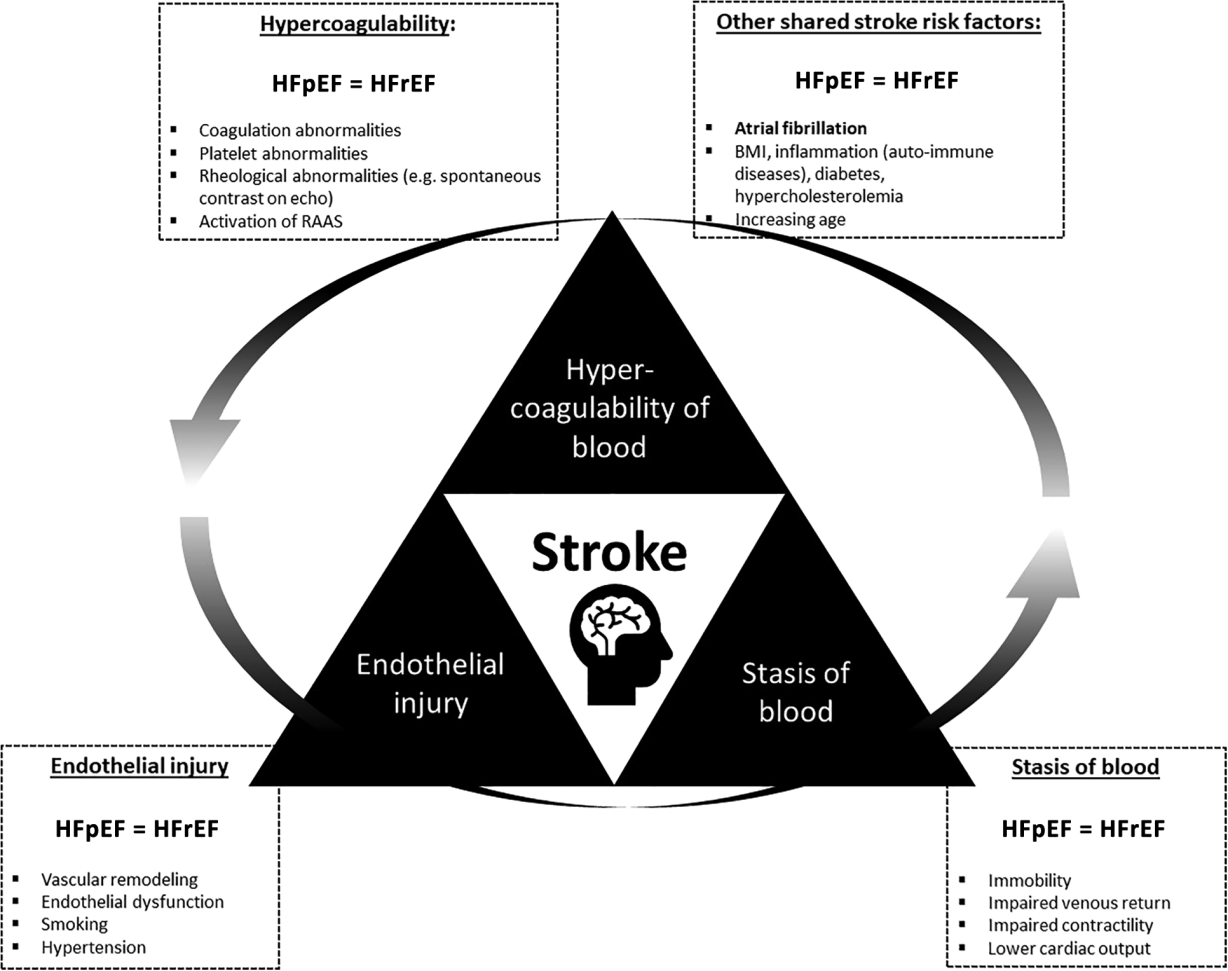
Hypothesis generating figure of the relation of stroke and heart failure. Illustrating that heart failure with preserved (HFpEF) and reduced ejection fraction (HFrEF) share similar risk factors for stroke using Virchow's triad. BMI, body mass index; RAAS, renin–angiotensin–aldosterone system.

For HFpEF and HFrEF the same pathophysiology processes are contributing to Virchow's pre‐requisites for thrombosis: abnormal blood flow, abnormalities in the blood vessel wall, and abnormal blood constituents (*Figure* *1*).^11^, ^12^ Constituent abnormalities are present in the form of abnormal platelets and increased levels of pro‐thrombotic markers.^11^ While the level of many circulating biomarkers increases with severity or worsening of heart failure, as is the case for e.g. the level of plasminogen activator inhibitor and tissue plasminogen activator antigen, both markers of fibrinolysis are elevated in heart failure patients across a wide range of LVEF, and regardless of LVEF.^11^, ^13^

## Stroke risk in heart failure regardless of left ventricular ejection fraction

The risk of stroke is significantly increased in patients with any reduction in LVEF and increases with a high CHA_2_DS_2_‐VASc score.^14^ The influence of LVEF on stroke risk appears to be substantial.^13^, ^15^ Recent data, however, suggest that the stroke risk is similarly increased in patients with reduced and preserved LVEF.^16^ A sub‐analysis of patients (from the non‐oral anticoagulation arm) participating in the Atrial Fibrillation Clopidogrel Trial With Irbesartan for Prevention of Vascular Events (ACTIVE) trials who also had heart failure were categorized as having preserved vs. reduced ejection fraction.^16^ Data from this study showed that the stroke risk was comparable between the two groups: 4.3% (in patients with HFpEF) and 4.4% (in HFrEF) per 100 person‐years.^16^ In addition, a meta‐analysis incorporating seven studies with a total of 33 773 patients with heart failure showed that for patients with HFrEF and HFpEF who also had AF the rate of stroke risk was similar at 1.6% in HFrEF and 1.3% in HFpEF (relative risk 0.85, *P* = 0.094).^15^

## Prevention of atrial fibrillation‐related stroke in heart failure regardless of left ventricular ejection fraction

**Figure 2:**
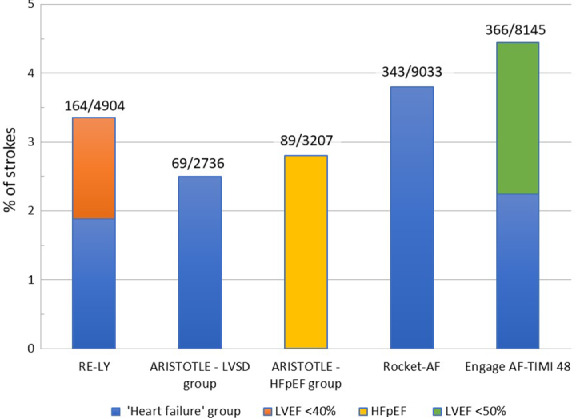
Percentages of stroke in heart failure patients in the four major novel oral anticoagulant trials. HFpEF, heart failure with preserved ejection fraction; LVEF, left ventricular ejection fraction; LVSD, left ventricular systolic dysfunction.

The most recent AF guidelines do not further differentiate the C (congestive heart failure) in the CHA_2_DS_2_‐VASc score, and score the ‘C’ when patients have signs/symptoms of heart failure or objective evidence of reduced LVEF. Indeed, there is no mention of HFpEF with regard to stroke prevention and as a result patients with HFpEF possibly must have more symptoms to receive anticoagulation (since they do not qualify with the LVEF criterium) than those with HFrEF. In the most recent heart failure guidelines it is stated that patients with heart failure (non‐specified) and in New York Heart Association (NYHA) functional class II–IV should be considered for anticoagulation, if eligible, as assessed by the CHA_2_DS_2_‐VASc score. Data on efficacy and safety of anticoagulation in heart failure patients have been published in several post‐hoc analyses of the landmark novel oral anticoagulant (NOAC) trials.^17^, ^18^, ^19^ In the heart failure substudy of the ROCKET‐AF trial, heart failure was defined as a history of heart failure (non‐specified) or a LVEF <40%.^17^ In the ARISTOTLE heart failure substudy, two groups of heart failure were defined. Patients with left ventricular systolic dysfunction (defined as LVEF <40%, or a documentation of moderate or severe left ventricular systolic dysfunction) with or without symptomatic heart failure. Or the second group which were heart failure patients who had symptomatic heart failure and LVEF >40%, normal left ventricular function, or mild left ventricular systolic dysfunction, grouped as HFpEF.^18^ In the RE‐LY trial, heart failure was defined as the presence of NYHA class II or higher in the 6 months before screening, in patients with a history of previous admission for congestive heart failure. Information about LVEF was available in only 2889 patients with heart failure (58.9%).^19^ A total 43.5% of the heart failure patients had a LVEF <40%, which may suggest that 56.5% of patients in the RE‐LY heart failure group had HFpEF (or that no measurement was available). In the ENGAGE AF‐TIMI 48 study, heart failure was defined as current presence or history of heart failure class C or D according to the American Heart Association/American College of Cardiology definition. In this study, 49% of patients had LVEF <50%, implying that half of the heart failure patients were HFpEF (of which many were classified as severe heart failure).^20^ *Figure* *2* shows the percentages of stroke in the heart failure groups and illustrates that in the heart failure population a significant proportion of patients had HFpEF. Overall the conclusions of these post‐hoc NOAC papers were that the effect of NOACs in patients with heart failure and AF is similar, both for efficacy as well as for safety outcomes as compared to AF patients without heart failure. Although one should be cautious to draw strong conclusions from the above studies in patients with HFpEF, these recommendations have in fact been made (from the historical data) for patients with HFrEF. ^17^, ^18^, ^19^, ^20^

## Summary

Given the recent increase in HFpEF and the fact that the CHA_2_DS_2_‐VASc is (mainly) based on HFrEF, criteria for anticoagulation for AF and HFpEF are in reality lacking. This is remarkable, given the fact that AF is more common in patients with HFpEF. However, as long as there are no trials performed specifically in this HFpEF population and there is no pathophysiological reason why data would be different in HFpEF, we believe that given the available data, anticoagulation must be seriously considered in many patients with AF and HFpEF. Indeed, recommendations for anticoagulation in AF/HFpEF patients may possibly be similar to those for HFrEF.

**Conflict of interest:** none declared.
